# Basketball training frequency is associated with executive functions in boys aged 6 to 8 years

**DOI:** 10.3389/fnhum.2022.917385

**Published:** 2022-07-22

**Authors:** Yue Xu, Wanxia Zhang, Kexin Zhang, Min Feng, Tianqi Duan, Yilin Chen, Xuexiang Wei, Yanlin Luo, Guoxin Ni

**Affiliations:** ^1^School of Sports Medicine and Rehabilitation, Beijing Sport University, Beijing, China; ^2^Department of Neurobiology, Capital Medical University, Beijing, China

**Keywords:** basketball, executive functions, frequency, children, boy

## Abstract

This study investigates the relationship between the frequency of basketball training and executive functions (inhibitory control, working memory, and cognitive flexibility) in boys aged 6 to 8. A total of 40 boys recruited from a local after-school basketball training club were divided into a low-frequency group (once a week) and a high-frequency group (at least twice a week). An additional 20 age-matched boys recruited from a local elementary school were considered as the control group (no training experience). All subjects conducted the Stop-signal task, the N-back task, and the switching task at rest. The mean reaction time and accuracy data obtained from each task were used in statistical analysis. There was no significant group difference in either the accuracy or reaction time of inhibitory control. Meanwhile, no significant difference was found in the reaction time of working memory across groups. However, the high-frequency group exhibited significantly higher accuracy (93.00 ± 4.31%) with regard to working memory than the low-frequency group (85.4 ± 6.04%, *P* < 0.001) and the control group (83.73 ± 7.70%, *P* < 0.001), respectively. A positive correlation was also found between the accuracy of working memory and groups. Furthermore, in comparison with the control group, the high-frequency group exhibited significantly higher cognitive flexibility accuracy (91.93 ± 7.40% vs. 85.70 ± 9.75%, *P* = 0.004) and shorter reaction time (934.24 ± 213.02 ms vs. 1,122.06 ± 299.14 ms, *P* < 0.001). There was also a positive correlation between the accuracy of cognitive flexibility and groups. These findings suggest that regular basketball training, especially with higher frequency, is beneficial to working memory and cognitive flexibilityin boys aged 6 to 8.

## Introduction

Executive Functions (EFs) refers to a set of high-level cognitive skills that control and regulate various basic cognitive processes when completing complex tasks (Miyake et al., 2000; Diamond, 2013). EFs are comprised of three main capacities: inhibition, working memory, and cognitive flexibility (Miyake et al., 2000). Inhibition relates to attention and behavior and prevents our acting underneath the manipulation of irrelevant environmental stimuli (Friedman and Miyake, 2017). Working memory refers to the ability to temporarily store information and utilize it for subsequent processing (Diamond, 2013). Cognitive flexibility is the ability to adapt our behavior correctly and efficiently in response to changes in the environment (Dajani and Uddin, 2015). The development of EFs is one of the most extensively researched elements of cognitive development during childhood and adolescence. Children’s academic performances (Espy et al., 2004), emotions (Blair, 2002), and social functioning (Espy et al., 2011), are affected by EFs, and these have also been shown to influence adult achievement in areas like health, income, and public safety (Moffitt et al., 2011). Therefore, monitoring EFs development during childhood is essential.

Sport is a physical activity that has defined goals, is governed by formal rules, and involves physical movement (Contreras-Osorio et al., 2021). Participation in sports offers multiple fitness advantages for children and adolescents, such as cognitive (Bidzan-Bluma and Lipowska, 2018), psychological (Eime et al., 2013), and musculoskeletal development (Krahenbühl et al., 2018) as well as decreased risk of obesity (Chen et al., 2020). Positive associations between physical activity and EFs have been reported (Li et al., 2020; Contreras-Osorio et al., 2021). As a worldwide popular sport, basketball belongs to the open skill sport category that covers a variety of sports forms such as running, jumping, and throwing, and involves a lot of skill acquisitions requiring high cognitive engagement in progress. Some researchers have also suggested that participating in cognitively-engaging physical activity (e.g., basketball) may have a stronger relationship with EFs than cognitively-engaging physical activity (e.g., running; Diamond and Ling, 2016; Ishihara et al., 2017a; de Greeff et al., 2018; Vazou et al., 2019).

The frequency of engaging in a sports activity can possibly be a vital component in the enhancement of EFs in children (Ishihara et al., 2017a). A previous study found that children who took tennis lessons once a week exhibited improved cognitive flexibility but no such development was found for inhibitory control or working memory (Ishihara et al., 2018). However, other studies have demonstrated that children who participated in physical activity interventions five times a week showed positive effects with regard to inhibitory control and working memory (Kamijo et al., 2011; Hillman et al., 2014). In parallel, additional research that focuses on the relationship between the frequency of physical activity and the development of EFs in children is needed.

The purpose of this cross-sectional study was to evaluate the association between the frequency of basketball training and three sub-components of EFs (inhibitory control, working memory, and cognitive flexibility). It was expected that children with high-frequency basketball training exhibit better EFs.

## Materials and Methods

### Participants

Sixty boys (aged 6–8) from a local elementary school and an after-school basketball training club were recruited to participate in the present study. Participants were categorized into three different groups based on the frequency of training: (1) control group: boys who only participate in the entertainment-oriented physical education lesson at school once a week, (2) low-frequency group: boys who participated in extra basketball training once a week, (3) high-frequency group: boys who participated in extra basketball training at least twice a week. All participants were right-handed and had normal or corrected visual acuity without color vision deficiencies or color blindness. Children who had physical or mental diseases, and cognitive or attention disorders and had participated in similar experiments were excluded from the study. All participants and their parents were informed about the purpose of this study. Parents or legal guardians provided written informed consent before the investigation. The study was approved by the Ethics Committee of Beijing Sport University. Table 1 shows participants’ demographic characteristics. The demographic profile was found to be comparable, with no statistically significant difference between the groups (*P* > 0.05).

**Table 1:** Participant demographics (Mean ± SD).

	**Control**	**Low-frequency**	**High-frequency**
N	20	20	20
Age (year)	7.4 ± 0.6	7.5 ± 0.7	7.7 ± 0.5
MMSE Score	29.0 ± 0.9	29.1 ± 0.8	29.3 ± 0.8
EHI Score	109.6 ± 14.0	120.6 ± 34.3	144.5 ± 69.1
CABI Score	4.4 ± 2.2	6.0 ± 2.3	3.6 ± 2.3
Training duration (month)	0	7.1 ± 1.9	7.6 ± 1.7

MMSE, Assessment of Mini-mental State Examination; EHI, Edinburgh Handedness Inventory; CABI, Child ADHD Behavior Inventory.

### Procedures

Prior to the test, subjects were screened using the Mini-Mental State Examination (MMSE), the Edinburgh Handedness Inventory (EHI), and the Attention-Deficit/Hyperactivity Disorder scale (ADHD). Only subjects who met all inclusion criteria were eligible to participate in the study. The tests were conducted between 10:00 and 13:00 in a quiet room, in which the temperature was maintained at about 23°C. Subjects performed the Stop-signal task, the N-back task, and the switching task to assess inhibitory control, working memory, and cognitive flexibility, respectively. To reduce error, three tasks were ordered randomly. The participants sat in front of a 17-inch computer screen (Lenovo ColorSync) that was about 57 cm away from their eyes. For stimulus presentation and data collection, E-Prime software (Psychology Software Tools, Inc. Pittsburgh, PA, USA) was used. Before the start of the formal experiment, practice experiments were conducted (same as the formal experiment). The formal experiment was carried out when the subjects fully grasped the experimental process, and the accuracy rate was 90% and above. Participants rested for 3–5 min between tasks. All measurements were evaluated at rest, after basketball training.

### Inhibitory control

The Stop-signal test was designed for assessing inhibitory control (Luo et al., 2021). This task consisted of GO and STOP trials. The GO stimulus is one of four black geometric figures (triangle, square, circle, and diamond), while the STOP stimulus is a black stop symbol. The experiment starts with a “+” prompt. A GO stimulus would show for 1,900 ms after a central fixation cross “+” appeared for 500 ms, sometimes followed by a STOP stimulus with stimulus onset asynchronies (SOAs) of 200, 400, 600, or 800 ms. Subjects were instructed to press the left button if the figure was not followed by a STOP symbol, and not click if the figure was followed by a STOP symbol. This experiment included a total of 208 trials, with 112 GO trials and 96 STOP trials (24 STOP trials for each SOA).

### Working memory

Working memory was assessed by the N-back task (Wolf et al., 2015). In the N-back test, participants were shown four figures (triangle, square, circle, and diamond) and are instructed to press a button when the current stimulus was the same as the item presented n-positions back. In this study, two blocks (the 0-back and 1-back tests) were administered. During the test, the screen would display with a black prompt “+” for 500 ms, reminding the participant to start the trial, and then one of the four figures would be displayed for 2,500 ms. For the 0-back block, participants had to press the left mouse button if the figure was a triangle, and press the right mouse button if not. For block 1-back, except for the first figure, participants needed to determine if the current figure was the same as the previous one and respond by pressing the left mouse button if it was the same or the right button if it is not. The figure would disappear automatically after clicking, then the screen would be blank for 500 ms before the next round of trials began. Participants had to respond within 3,000 ms otherwise the answer to this trial would be recorded as an error. Each block had 80 trials, and each trial took 3,000 ms.

### Cognitive fexibility

We used the Switching task to estimate cognitive flexibility (Luo et al., 2021). The stimulus in this experiment was two of the four figures (triangle, square, circle, and diamond), that were placed horizontally and highlighted in red and green, respectively. A green (or red) central “+” was displayed as a cue for 1,000 ms, followed by the appearance of a stimulus for 3,000 ms. The target was assumed to be the figure with the same color as the cue. If the target was (or was not) a triangle figure, each participant was asked to click the left (or right) mouse key. After pressing the button, the stimulus would disappear automatically, and after 500 ms of blank, the next trial would start. There were 186 trials in this experiment, which were divided into three blocks. The first was the sustained condition, which contained two consecutive trials and two cues of the same color. The second was the switching condition, which contained two consecutive trials but the two cues were of different colors. The third was the sustained between switching condition, which had three or more consecutive trials and the cues are in two colors.

### Statistical analysis

The statistical analysis was performed with SigmaStat 3.5 (SigmaStat 3.5, Erkrath, Germany). Prior to the analysis, the assumption of normality was checked using the Shapiro–Wilk test. The demographic characteristics of the three groups were compared using one-way ANOVA. The accuracy data obtained from the STOP trial of Stop-signal task was analyzed using 3 (group: control, low-frequency, high-frequency) × 4 (SOAs: 200 ms, 400 ms, 600 ms, 800 ms) two-way ANCOVAs. For the mean RT data obtained from the GO trial of Stop-signal task, one-way ANOVA was performed. The mean RT and accuracy data obtained during the other two EFs tasks were analyzed using 3 (group: control, low-frequency, high-frequency) × 2 (block) two-way ANCOVAs. Significant main effects of the groups were further analyzed *via* the Holm-Sidak *post-hoc* test. Correlations between the mean RT or accuracy data of the EFs task and groups were performed by calculating Spearman rank correlation coefficients (*r*). Correlation coefficient strength was classified as negligible <0.30, weak 0.31–0.50, moderate 0.51–0.70, or strong >0.71 (Sonesson et al., 2021). The significance level was set at *P* = 0.05.

## Result

### Stop-signal task

**Figure 1:**
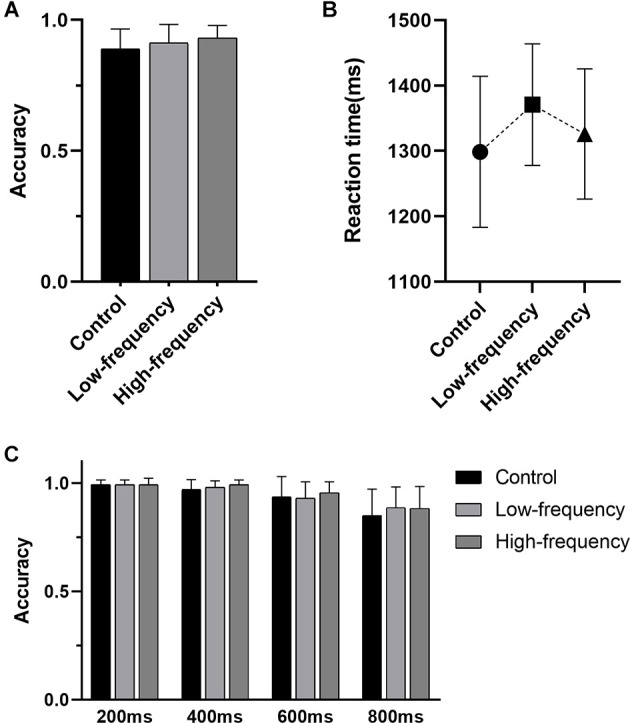
Results of the Stop-signal task. **(A)** The accuracy of the GO trials, **(B)** the reaction time of the Go trials, **(C)** the accuracy of the STOP trials with different stimulus onset asynchronies.

For the STOP trial of Stop-signal task, significant Group × SOAs interaction effects were not present (*F*_(6,228)_ = 0.524, *P* = 0.790). There was no significant main effect of group (*F*_(2,228)_ = 1.601, *P* = 0.204) but there was a significant main effect of SOAs (*F*_(3,228)_ = 37.309, *P* < 0.001). However, between-group analyses showed no significant difference (Figure 1). No significant correlation was apparent between group and SOAs (Table 2). For the GO trial of the Stop-signal task, statistical analysis of the accuracy and RT showed no significant difference between groups (Figure 1), meanwhile correlation was not found (Table 3).

**Table 2:** The accuracy of the Stop Trials at different interval times in each group (Mean ± SD%).

**SOAs (ms)**	**Control**	**Low-frequency**	**High-frequency**	***r* (*p* value)**	
200	99.30 ± 2.10	99.30 ± 2.10	99.30 ± 3.00	0.066 (0.614)
400	97.00 ± 4.60	98.00 ± 3.10	99.30 ± 2.10	0.250 (0.054)
600	93.70 ± 9.30	93.00 ± 7.60	95.60 ± 5.00	-0.002 (0.989)
800	85.00 ± 12.20	88.70 ± 9.50	88.30 ± 10.10	0.082 (0.534)

SOAs, stimulus onset asynchronies.

**Table 3:** The accuracy and reaction time of the Go Trials in each group (Mean ± SD).

**Indicators**	**Control**	**Low-frequency**	**High-frequency**	***r* (*p* value)**	
Accuracy (%)	89.60 ± 7.60	91.20 ± 7.12	93.10 ± 4.75	0.239 (0.066)
Reation time (ms)	1,298.65 ± 115.64	1,370.80 ± 93.13	1,325.95 ± 99.55	0.105 (0.425)

### N-back task

**Figure 2:**
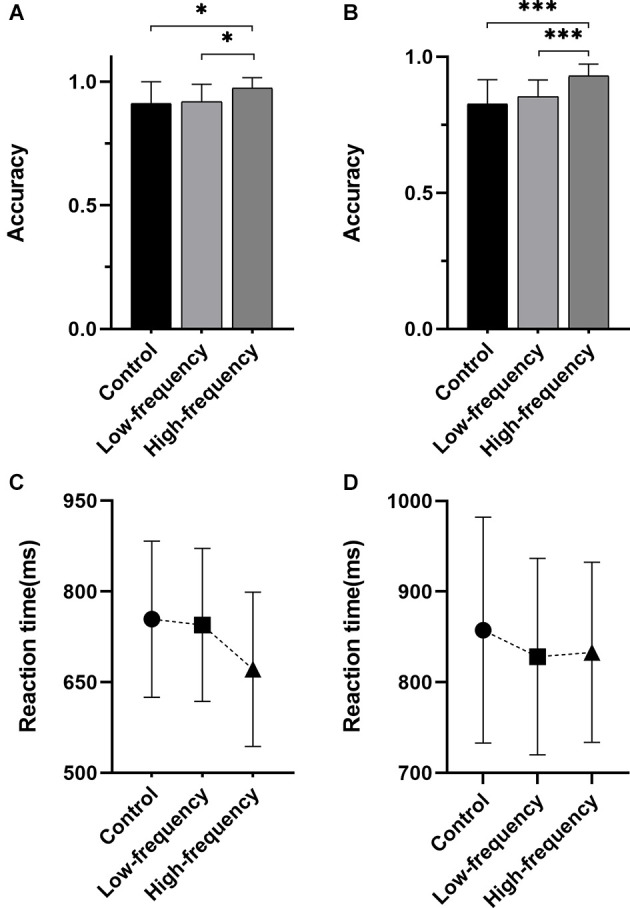
Results of the N-back task. **(A)** The accuracy of the 0-back task, **(B)** the accuracy of the 1-back task, **(C)** the reaction time of the 0-back task, **(D)** the reaction time of the 1-back task. *Statistically significant with *P* < 0.05, ***statistically significant with *P* < 0.001.

In the case of the accuracy of N-back task, no significant interaction effect existed between group and block (0-back task, 1-back task; *F*_(2,113)_ = 0.594, *P* = 0.554). Otherwise, there was a significant main effect of group (*F*_(2,113)_ = 15.939, *P* < 0.001) and a significant main effect of block (*F*_(1,113)_ = 25.067, *P* < 0.001). Intergroup analysis showed that the accuracy of the high-frequency group was significantly higher than the other two groups in the 0-back task and 1-back task (*P* < 0.05, Figure 2), There was a weak positive correlation between the accuracy of 0-back task and groups (*r* = 0.317, *P* = 0.014); meanwhile, a moderate positive correlation was found between the accuracy of 1-back task and groups (*r* = 0.538, *P* < 0.001, Table 4). In terms of the reaction time, there was no significant interaction effect between group and block (*F*_(2,113)_ = 1.417, *P* = 0.247); meanwhile, while there was no significant main effect of the group (*F*_(2,113)_ = 1.509, *P* = 0.225), there was a significant main effect of block (*F*_(1,113)_ = 25.307, *P* < 0.001). Between groups analysis showed no significant difference in reaction time throughout groups (Figure 2). There was a negligible negative correlation between the reaction time and groups (*r* = -0.0273, *P =* 0.035, Table 4).

**Table 4:** The accuracy and reaction time of the N-back Task in each group (Mean ± SD).

**Block**	**Indicators**	**Control**	**Low-frequency**	**High-frequency**	***r* (*p* value)**	
0-back	Accuracy (%)	91.13 ± 8.78a*	91.80 ± 7.00a*	97.30 ± 4.27	0.317 (0.014)*
	Reaction time (ms)	754.11 ± 129.04	744.55 ± 126.50	671.24 ± 127.50	−0.273 (0.035)*
1-back	Accuracy (%)	83.73 ± 7.70a***	85.40 ± 6.00a***	93.00 ± 4.31	0.538 (0.000)***
	Reaction time (ms)	838.26 ± 115.00	828.23 ± 108.41	832.96 ± 99.46	−0.152 (0.246)

*Statistically significant with *P* < 0.05, ***statistically significant with *P* < 0.001, ^a^compared with the high-frequency group.

### Switching task

**Figure 3:**
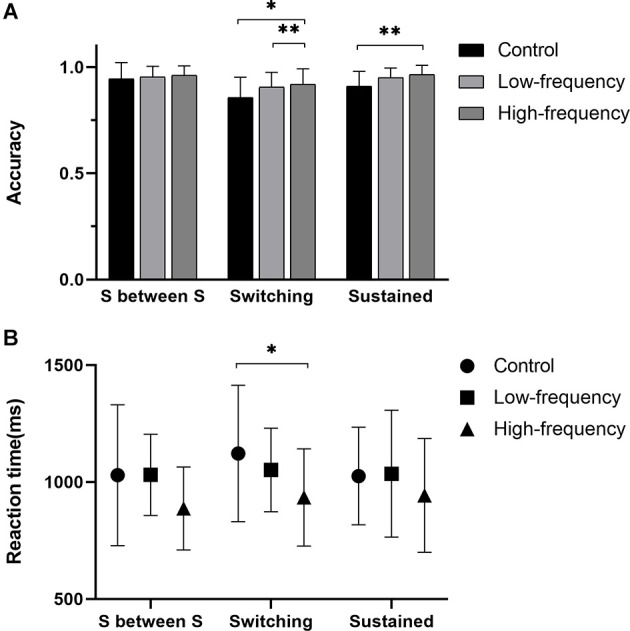
Results of the Switching task. **(A)** The accuracy of three blocks, **(B)** the reaction time of three blocks. S between S for Sustained between Switching block. *Statistically significant with *P* < 0.05, **statistically significant with *P* < 0.01.

As far as the accuracy of the Switching task, there was no significant interaction effect between group and block (Sustained between Switching, Switching, Sustained) (*F*_(4,171)_ = 0.833, *P* = 0.506). Nevertheless, there was also a significant main effect of group (*F*_(2,171)_ = 7.143, *P* = 0.001) and a significant main effect of block (*F*_(2,171)_ = 13.048, *P* < 0.001). Intergroup analysis showed that the accuracy of the control group was significantly lower than that of the other two groups (*P* < 0.005, Figure 3) in the Switching block, while in Sustained block, the accuracy of the control group was significantly lower than that of the high-frequency group (*P* = 0.009, Figure 3). There was a negligible positive correlation between the accuracy of the Switching block and groups (*r* = 0.254, *P* = 0.049), and a weak positive correlation between the accuracy of Sustained block and groups was found (*r* = 0.332, *P* = 0.010, Table 5). In terms of the reaction time, significant Group × Type of task interaction effects were not present (*F*_(4,171)_ = 0.314, *P* = 0.868). There was no significant main effect on type of task (*F*_(2,171)_ = 0.778, *P* = 0.461) but here was a significant main effect of group (*F*_(2,171)_ = 5.818, *P* = 0.004). Between groups analysis showed the reaction time of the high-frequency group was significantly lower than that of the control group in the Switching task (*P* = 0.014, Figure 3). However, no significant correlation was discovered (Table 5).

**Table 5:** The accuracy and reaction time of the Switching Task in each group (Mean ± SD).

**Block**	**Indicators**	**Control**	**Low-frequency**	**High-frequency**	***r* (*p* value)**	
Sustained between Switching	Accuracy (%)	94.50 ± 7.80	95.24 ± 5.21	96.11 ± 4.47	0.034 (0.798)
	Reaction time (ms)	1,029.23 ± 309.44	1,030.89 ± 178.00	886.80 ± 182.07	−0.190 (0.146)
Switching	Accuracy (%)	85.70 ± 9.75	90.53 ± 7.15a*	91.93 ± 7.40a**	0.254 (0.049)*
	Reaction time (ms)	1,122.06 ± 299.14	1,051.80 ± 183.61	934.24 ± 213.02a*	−0.283 (0.029)
Sustained	Accuracy (%)	90.80 ± 7.30	95.00 ± 4.64	96.40 ± 4.50a**	0.332 (0.010)*
	Reaction time (ms)	1,025.66 ± 214.07	1,035.73 ± 278.27	943.05 ± 249.42	−0.196 (0.134)

*Statistically significant with *P* < 0.05, **statistically significant with *P* < 0.01, ^a^compared with the control group.

## Discussion

In this study, three cognitive tests were administered to children who were undergoing three different frequencies of training to understand the differences and relationships between basketball training frequency and EFs of 6–8-year-old boys. It was found only higher frequency basketball training can enhance the EFs of children aged 6–8, especially with regard to cognitive flexibility and working memory.

The results of this study showed that the frequency of basketball training was positively associated with the enhancement of EFs in children. Training frequency is a crucial element affecting the relationship between physical activity and EFs (Ishihara et al., 2017a). In accordance with the present results, previous studies have demonstrated that weekly open skills training had no significant impact on children’s inhibitory control and working memory (Ishihara et al., 2018), while prolonged training of the same type five times a week improved their cognitive flexibility, inhibitory control, and working memory (Hillman et al., 2014). Basketball is a cognitively engaging exercise that requires the understanding of rules, peer competition, and the memory of technical movements, all of which require the participation of EFs (Rösch et al., 2021). It can be concluded that increasing the frequency of a cognitively engaging exercise has a positive effect on children’s EFs. This may be attributable to a dose-response relationship between the frequency and EFs (Ishihara et al., 2017b).

Another important finding was that increasing the frequency of basketball training can enhance EFs of children, especially cognitive flexibility and working memory, which accords with earlier observations (Bryant et al., 2021). Cognitive flexibility is the ability to shift attention and focus on accomplishing an internal goal or fulfilling task demands (Garon et al., 2008). In the case of basketball, a high level of concentration is necessary to ensure rhythmic follow-through while dribbling the ball. Also, basketball as an open skill requires participants to perform in a dynamic environment and respond to unpredictable and frequent environmental changes throughout the activity (Dai et al., 2013; Chiu et al., 2020; Ke et al., 2021). Therefore, basketball training will engage children in quickly evaluating all possible situations to choose a preferred strategy and switching technical actions when necessary (Pedro et al., 2021), it is possible that increasing the frequency of basketball training allows children to practice their cognitive flexibility (Nuri et al., 2013). Working memory refers to the ability to remember information while keeping it accessible for use (Baddeley et al., 1986). Possible explanations for increasing the frequency of basketball training to improve children’s working memory might be that on the one hand, when children are participating in basketball training, they are expected to continually keep the basketball rules in mind which likely elicits working memory throughout participation. On the other hand, during the basketball training, children need to maintain, update, and extract the information related to the task objectives is required, while simultaneously ignoring or suppressing the competitive information irrelevant to the current situation, so effective information processing needs the participation of working memory (Furley and Memmert, 2012; Vaughan and Laborde, 2021). More subtly, we found significant advantages in the accuracy of working memory and cognitive flexibility and reaction time of switching for children in the high-frequency training group. These advantages in accuracy may be related to the fact that basketball is a goal-oriented sport and that shooting accuracy is emphasized in training. After all, shooting accuracy is one of the most crucial talents in determining basketball success, and it’s a key aspect in determining which basketball teams win and which lose (Trninić et al., 2002; Chen et al., 2018). Moreover, children in the high-frequency training group were faster at switching reactions, probably because in the dynamic training of passing the ball between marches, participants are asked to exclude extraneous information (e.g., audience chants, distractions caused by opponents), quickly find strategies, and switch movements in time, thus greatly improving their ability to switch.

This result may be explained by the fact that physical activity improves EFs seems to be associated with the physiological changes it causes in the brain. Studies have proved that regular participation in physical activity has been correlated with positive changes to brain structure and volume, such as an increase in white matter, parietal lobe gray matter, hippocampal, and basal ganglia volume (Erickson et al., 2009; Benedict et al., 2013; Niemann et al., 2014). In addition, physical activity is considered to influence brain neuroplasticity because it increases brain-derived neurotropic factor (BDNF) synthesis in the hippocampus, promotes neuronal and synaptic growth and differentiation, and protects neuronal and synaptic transmission (Lista and Sorrentino, 2010). Moreover, a recent study proved that exercise can improve blood circulation to the brain, and clustering in exercise plasma reduces inflammation in the brain and improves memory (De Miguel et al., 2021). These effects may be more pronounced in children, as motor and cognitive skills have a similar developmental timeline—both accelerate between the ages of 6 and 12. At this time, their brains are developing rapidly, especially in the dorsolateral prefrontal cortex, anterior cingulate cortex, parietal cortex, and subcortical structures, such as the thalamus, caudate nucleus, putamen, and cerebellum (Bidzan-Bluma and Lipowska, 2018). As such, we suggest that children in this age group should increase physical activity to achieve better motor-cognitive benefits (Álvarez-Bueno et al., 2017).

However, unlike the results for working memory and cognitive flexibility, our study did not find differences in inhibitory control between children with different frequencies of basketball training. Several reasons may explain this outcome. First, the three EFs components may be independent of one another (Welsh et al., 1991), and their progression is age dependent (Huizinga et al., 2006; Best and Miller, 2010). In addition, using the Stop-signal task to measure inhibitory control will reveal an older age advantage (Cragg and Nation, 2008). Due to the age of the participants in this study, they might not have had sufficient cognitive development to fully comprehend the task they were asked to complete. Second, the type of assessment and activity may have affected the relationship between children’s inhibition and physical activity (Burkart et al., 2018; Ludyga et al., 2022). Our studies trained children on a schedule that did not add interactive competition-based training, so the less complex physical activity task may not have provided sufficient stimulation to improve inhibitory control.

Although our study confirms that children with higher basketball training frequency have better working memory and cognitive flexibility and also reveals a positive dose-response relationship between exercise and EFs, several limitations should be acknowledged. First, this study used a cross-sectional design, thus we could not confirm the causal effects. Second, children’s cognitive development is influenced by age and gender (Ishihara et al., 2018), but our study was conducted only with boys aged 6–8, and thus the results may not be generalizable to girls or other age groups. Third, we only looked at the dose-response effect of basketball training, so we can not compare it to other sports and there may be a possible uncontrolled effect of preexisting preferences. Fourth, even if all three groups had the same physical activity exposure frequency at school but it is not clear whether different school-based physical education types could affect children’s EFs. In the end, all our studies yielded behavioral outcomes, so we could not determine the physiological causes of task performance changes. Longitudinal studies or intervention designs and more diverse measures (e.g., neuroimaging) are needed to confirm that cognitively engaging exercise is a useful tool for supporting the development of EFs in children.

In conclusion, more frequent basketball training may contribute to the development of working memory and cognitive flexibility in boys aged 6–8 but not to inhibitory control.

## Data Availability Statement

The raw data supporting the conclusions of this article will be made available by the authors, without undue reservation.

## Ethics Statement

The studies involving human participants were reviewed and approved by the Ethics Committee of Beijing Sport University (2022072H). Written informed consent to participate in this study was provided by the participants’ legal guardian/next of kin.

## Author Contributions

YX and WZ wrote the manuscript. YX analyzed the data. KZ, MF, TD, YC, and XW recruited subjects, carried out the experiments, and collected the data. YL conceived and planned the experiments, and GN made critical revisions related to important intellectual content of the manuscript. All authors contributed to the article and approved the submitted version.

## Conflict of Interest

The authors declare that the research was conducted in the absence of any commercial or financial relationships that could be construed as a potential conflict of interest.

## Publisher’s Note

All claims expressed in this article are solely those of the authors and do not necessarily represent those of their affiliated organizations, or those of the publisher, the editors and the reviewers. Any product that may be evaluated in this article, or claim that may be made by its manufacturer, is not guaranteed or endorsed by the publisher.
